# Successful endoscopic ultrasound-guided choledochoduodenostomy using a novel double-lumen dilator

**DOI:** 10.1055/a-2849-9128

**Published:** 2026-04-27

**Authors:** Tsuyoshi Suda, Haruki Yamagishi, Taiga Saito, Norihiko Ogawa, Yoshihide Naito, Kenkei Hasatani, Hiroyuki Aoyagi

**Affiliations:** 1 Department of Gastroenterology 13607 Fukui Prefectural HospitalFukui CityFukui PrefectureJapan

**Fig. 1:**
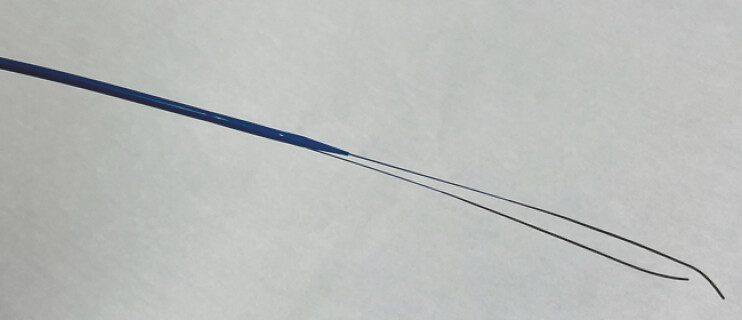
A novel double-lumen tapered dilator (MEISSA; Japan Lifeline, Tokyo, Japan).

Endoscopic ultrasound (EUS)-guided biliary drainage (BD) occasionally requires secure guidewire manipulation and stable tract dilation 1 . A newly developed double-lumen tapered dilator (MEISSA; Japan Lifeline, Tokyo, Japan; Fig. 1 ) allows contrast injection and guidewire handling using the same device, thus facilitating rapid transition to the double-guidewire technique without the need for catheter exchange.

**Fig. 2:**
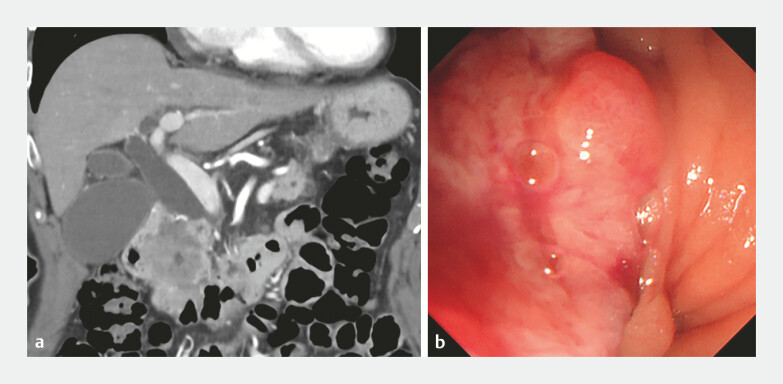
**a** A contrast-enhanced computed tomographic image of pancreatic head cancer with malignant biliary stenosis and **b** an endoscopic image of duodenal invasion.

An 83-year-old woman with pancreatic head cancer ( Fig. 2 **a** ) presented with malignant biliary obstruction. Endoscopic retrograde cholangiopancreatography with biliary stent placement was attempted; however, the procedure was unsuccessful because of duodenal invasion ( Fig. 2 **b** ). Therefore, EUS-guided choledochoduodenostomy (CDS) was performed. After puncturing the common bile duct using a 19-G EUS fine-needle biopsy needle, the initial guidewire was placed. Nonetheless, selective access to the intrahepatic bile duct was difficult.

**Fig. 3:**
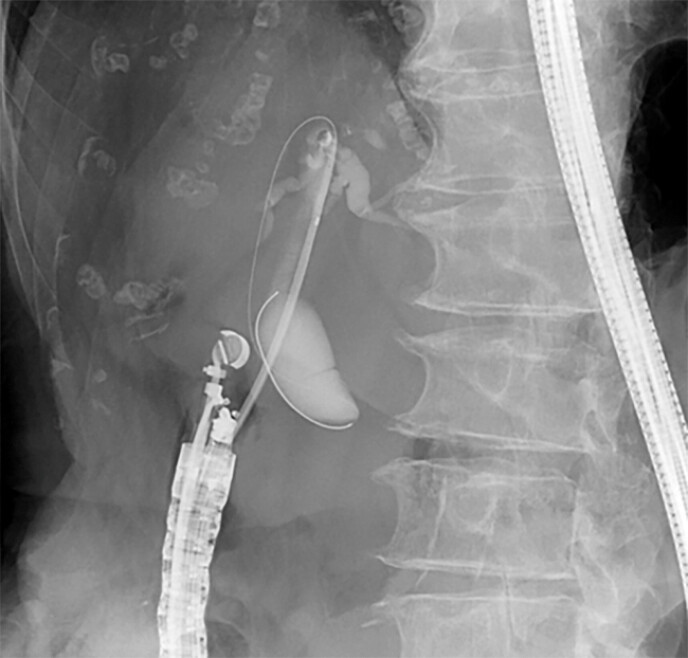
The novel double-lumen dilator enabled contrast injection through the second lumen and maintained the placement of the first guidewire.

**Fig. 4:**
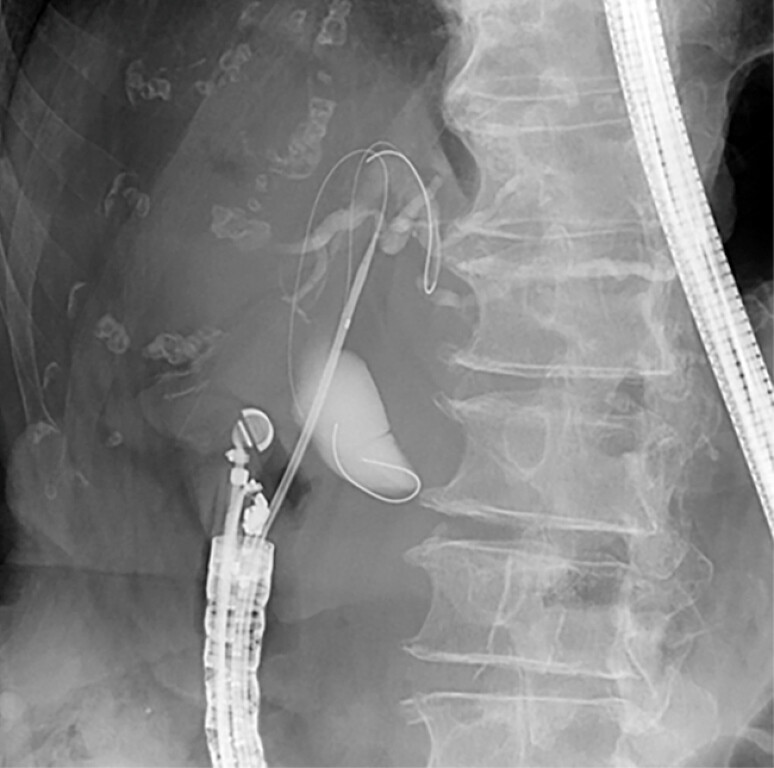
Successful advancement and placement of the second guidewire in segment B3.

**Fig. 5:**
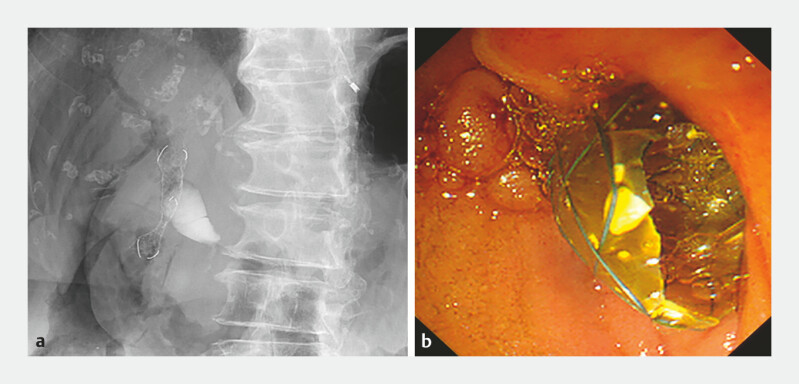
**a, b** Successful deployment of a fully covered metal stent.

The puncture tract was dilated using the novel double-lumen dilator. Contrast injection through the lumen without the initial guidewire was performed to visualize the intrahepatic bile ducts ( Fig. 3 ). Subsequently, a second guidewire was inserted, and selective cannulation of segment B3 was successfully performed ( Fig. 4 ). This dilator enabled these steps without the need for device exchange ( Video 1 ). Finally, a fully covered metal stent was deployed with stable guidewire support ( Fig. 5 **a, b** ). The procedure was completed successfully without adverse events.

Contrast injection and placement of the second guidewire are performed using the novel double-lumen dilator.Video 1

During interventional EUS, the double-guidewire technique improves technical success rates and procedural safety 2 . The dual-lumen design reduces the number of device insertions, minimizes the risk of guidewire displacement, and improves procedural stability. These are important advantages in technically demanding interventional EUS-BD cases. Moreover, it allows a seamless transition to the double-guidewire technique when required. Several reports of EUS-BD using this device have been published 3 4 . This novel dilator may streamline the workflow, shorten procedure times, and enhance the technical success of EUS-BD with EUS-CDS.

Endoscopy_UCTN_Code_TTT_1AS_2A
